# Liposomal Antioxidants for Protection against Oxidant-Induced Damage

**DOI:** 10.1155/2011/152474

**Published:** 2011-08-16

**Authors:** Zacharias E. Suntres

**Affiliations:** Medical Sciences Division, Northern Ontario School of Medicine, Lakehead University, 955 Oliver Road, Thunder Bay, ON, Canada P7B 5E1

## Abstract

Reactive oxygen species (ROS), including superoxide anion, hydrogen peroxide, and hydroxyl radical, can be formed as normal products of aerobic metabolism and can be produced at elevated rates under pathophysiological conditions. Overproduction and/or insufficient removal of ROS result in significant damage to cell structure and functions. *In vitro* studies showed that antioxidants, when applied directly and at relatively high concentrations to cellular systems, are effective in conferring protection against the damaging actions of ROS, but results from animal and human studies showed that several antioxidants provide only modest benefit and even possible harm. Antioxidants have yet to be rendered into reliable and safe therapies because of their poor solubility, inability to cross membrane barriers, extensive first-pass metabolism, and rapid clearance from cells. There is considerable interest towards the development of drug-delivery systems that would result in the selective delivery of antioxidants to tissues in sufficient concentrations to ameliorate oxidant-induced tissue injuries. Liposomes are biocompatible, biodegradable, and nontoxic artificial phospholipid vesicles that offer the possibility of carrying hydrophilic, hydrophobic, and amphiphilic molecules. This paper focus on the use of liposomes for the delivery of antioxidants in the prevention or treatment of pathological conditions related to oxidative stress.

## 1. Introduction

**Figure 1:**
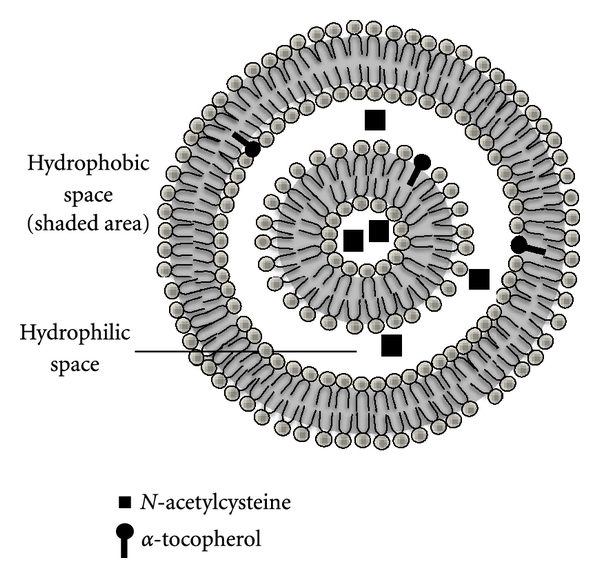
Schematic representation of a liposome containing antioxidants. Liposomes are artificially prepared vesicles made of lipid bilayer. Water-soluble compounds (e.g., N-acetylcysteine (NAC)) can be encapsulated in the aqueous phase, while lipid-soluble compounds (e.g., *α*-tocopherol) can be incorporated into the lipid bilayer of the liposome.

Oxidative stress (OS) is defined as an imbalance between the production of reactive oxygen species and antioxidant defenses that can lead to cellular and tissue damage [1–8]. A potential pharmacological strategy in preventing or treating oxidant-induced cellular and tissue damage involves the use of appropriate antioxidants. Antioxidants are substances which are able to prevent, delay, or remove oxidative damage to a molecule [4, 9–11]. Yet, their efficacy is hindered with challenges such as poor solubility, inability to cross cell-membrane barriers, extensive first-pass metabolism, and rapid clearance of antioxidants from cells [12, 13]. To improve the pharmacological and pharmacokinetic properties of antioxidants, diverse systems such as antioxidant chemical modifications and coupling to affinity carriers, micelles, and liposomes are being developed [4, 13–18]. This paper focus on the use of liposomes for the delivery of antioxidants in the prevention or treatment of several pathological conditions linked to oxidative stress. Liposomes are artificial vesicles consisting of an aqueous core enclosed in one or more phospholipid layers; water-soluble compounds can be encapsulated in the aqueous core, while lipid-soluble compounds can be incorporated into the lipid bilayer of the liposome (Figure 1) [19–21]. Encapsulation of enzymatic antioxidants (superoxide dismutase and catalase) or nonenzymatic antioxidants (glutathione, N-acetylcysteine, CoQ10, curcumin, resveratrol, *α*-tocopherol, and *γ*-tocopherol) in liposomes improves their therapeutic potential against oxidant-induced tissue injuries, because liposomes apparently facilitate intracellular delivery and prolong the retention time of entrapped agents inside the cell.

## 2. Oxidative Stress and the Antioxidant Defense System

The involvement of oxidants in several pathological disorders, including cancer, diabetes, cardiovascular diseases, chronic inflammatory disease, postischaemic organ injury, neurodegenerative disorders, and xenobiotic/drug toxicity has been widely accepted, but whether such oxidants are the major cause of tissue injury in human disease or simply produced during the development of injury is still under debate [1–8]. Regardless, the interaction of reactive oxygen species (ROS) with macromolecules, organelles, and tissues results in damage. For example, membrane lipid oxidation may result in an irreversible injury to the plasma membrane [22]. Protein oxidation may lead to a loss of critical sulphydryl groups in addition to modifications of amino acids leading to the formation of carbonyl and other oxidized moieties [23]. Attack of ROS on nucleic acids can generate various modified bases in DNA which play a critical role in carcinogenesis and aging [1]. 

In general, oxidative stress describes the result of an increased ROS production and/or a decrease in their elimination [24]. Such ROS include, but certainly not limited to, superoxide anion, hydrogen peroxide, hydroxyl radical, and lipid peroxides [25, 26]. Some of these species, such as superoxide or hydroxyl radicals, are extremely unstable, whereas others, like hydrogen peroxide, are freely diffusible and relatively long lived. The sources of ROS may be exogenous or endogenous [2, 11]. During their metabolism, agents such as paraquat or doxorubicin generate ROS resulting in pneumotoxicity or cardiotoxicity, respectively [4, 27]. Intrinsically generated oxidants may be derived from the electron transport chain in the mitochondria, with the most important source of damaging oxidants being the phagocytic cells (residential macrophages and recruited neutrophils) that can generate ROS and reactive nitrogen species from the NADPH oxidase assembly located on their cell surfaces [2, 28, 29]. Other pathways that may participate in the formation of ROS include xanthine oxidase in ischemia-reperfusion and cytochrome P-450-dependent activation of xenobiotics [11, 26]. 

Sequential reduction of molecular oxygen leads to the formation of superoxide anion, hydrogen peroxide, and hydroxyl radical. The univalent reduction of molecular oxygen forms superoxide anion in a reaction mediated by enzymes such as xanthine oxidase and NAD(P)H oxidase or nonenzymatically by redox-reactive compounds such as the semiubiquinone compound of the mitochondrial electron transport chain [11, 26, 30]. Superoxide dismutases convert the superoxide anion into hydrogen peroxide which in the presence of reduced transition metals (e.g., ferrous or cuprous ions) is metabolized to the highly reactive hydroxyl radical [30]. Other reactive species include nitric oxide, lipid radicals, peroxynitrite, and hypochlorous acid [11, 29]. Nitric oxide is synthesized from the guanidine group of L-arginine by a family of enzymes termed nitric oxide synthases. Simultaneous generation of nitric oxide and superoxide anion favors the production of a strong oxidant, the peroxynitrite anion, which may account for some of the deleterious effects associated with nitric oxide production [31]. All these reactive oxygen species are potentially very reactive molecules and, at high enough concentration, result in damage to critical cellular constituents (such as proteins, nucleic acids, carbohydrates, and lipids) resulting in cell necrosis [11, 26, 28, 29]. 

At physiological low levels, ROS function as “redox messengers” in intracellular signaling and regulation. Redox signaling is a well-recognized stress response that leads to a variety of downstream effects including increased expression of protective and repair enzymes [32, 33]. There is also mounting evidence that redox signaling is part of normal metabolism in nonstressed cells. In this situation, endogenously generated oxidants act as second messengers for receptor agonists such as growth factors and hormones, signaling the proliferative or metabolic changes associated with these ligands [32, 33]. Oxidants can activate and inactivate transcription factors, membrane channels, and metabolic enzymes, and modulate calcium-dependent and phosphorylation signaling pathways. These processes incorporate the major regulatory networks of cells, giving redox signals the capacity to stimulate and adjust most aspects of cell physiology [34].

Exposure to reactive oxygen species from a variety of sources has led organisms to develop a series of defense mechanisms [9, 35]. The harmful effects of ROS are counterbalanced by the antioxidant action of both antioxidant enzymes and nonenzymatic antioxidants [30]. Antioxidants have been defined as substances that are able to prevent, delay, or remove oxidative damage to a molecule [36] and may be categorized in the following groups: (i) those aimed at preventing the generation and distribution of reactive oxygen and nitrogen species (e.g., the effective control of iron distribution and the destruction of peroxides by catalase or by glutathione peroxidase), (ii) those aimed at reactive metabolite scavenging including the maintenance of effective levels of antioxidants, such as vitamin E, vitamin C, *β*-carotene, and glutathione as well as the enzyme superoxide dismutase, and (iii) those aimed at free radical repair, particularly the maintenance of effective levels of glutathione [4, 10, 11, 36]. 

The antioxidant enzymes found in living cells include the superoxide dismutase, catalase, glutathione peroxidase, peroxiredoxins, and thioredoxin reductase [4, 10, 11, 13, 25, 36–38]. These antioxidant enzymes are not consumed during their catalytic actions, and they have high affinity and rate of reaction with ROS consequently allowing more effective protection against acute massive oxidative insults. Superoxide dismutases (SODs) are metalloenzymes that catalyze the conversion of superoxide anion to hydrogen peroxide and include the cytosolic copper and zinc containing form (Cu-Zn-SOD), the mitochondrial manganese-containing SOD (MnSOD), and an extracellular form (EC-SOD) [39]. Catalase, which catalyzes the detoxication of hydrogen peroxide to water and oxygen, occurs abundantly in the body, with the highest activity in the liver, followed by erythrocytes, then the lungs [13, 25]. Glutathione peroxidase plays a major role in the detoxication of hydrogen peroxide, other hydroperoxides, and lipid peroxides via the glutathione redox cycle [11, 25]. The thioredoxin system, consisting of thioredoxin reductase in conjunction with thioredoxin and NADPH, is a ubiquitous oxidoreductase system with antioxidant and redox regulatory roles; mammalian thioredoxin reductase has a highly reactive active site selenocysteine residue resulting in a profound reductive capacity, reducing several substrates in addition to thioredoxin [37]. Peroxiredoxins are ubiquitous thiol-dependent peroxidases that accomplish the same function as other antioxidant enzymes such as catalase and glutathione peroxidase, but their catalytic activity is lower than that of these enzymes. Peroxiredoxin 6 is a bifunctional protein with both GSH peroxidase and phospholipase A_2_ activities that play important physiological roles in antioxidant defense and lung surfactant metabolism [38, 40].

Nonenzymatic antioxidative systems are not as specific as enzymatic ones, but they serve as the first line of defense and are, therefore, of high importance in cellular response to oxidative stress. The major nonenzymatic antioxidants include vitamin C (ascorbic acid), vitamin E (tocopherols and tocotrienols), glutathione, carotenoids, flavonoids, and other micronutrients. Vitamin C, a water-soluble vitamin, is effective in scavenging ROS, including hydroxyl radical, aqueous peroxyl radicals, and superoxide anion [10]. Vitamin E, the principal antioxidant in the body, is composed of four tocopherols and four tocotrienols, and, due to its lipophilicity, it is present in all cellular membranes [41]. The antioxidant functions of vitamin E include the termination of chain reactions in polyunsaturated fatty acids in cell membranes from peroxidation by scavenging peroxyl radicals in membranes and neutralizing the highly reactive singlet oxygen molecules [42]. Glutathione (GSH) is the most abundant nonprotein thiol in living organisms and can exert its protective effects by acting as a nucleophile to form conjugates with many xenobiotic compounds and/or their metabolites and serve as a reductant in the metabolism of hydrogen peroxide and other organic hydroperoxides, a reaction catalyzed by glutathione peroxidases found in cytosols and mitochondria of various cells [43, 44]. In humans, a number of neurodegenerative and neuropsychiatric conditions are associated with disturbances in glutathione [45]. Repletion and maintenance of neuronal GSH is, therefore, important to cell health and viability and could provide therapeutic benefit in situations when GSH is deficient. Carotenoids are a class of natural fat-soluble pigments found principally in plants, the most abundant in the diet being beta-carotene, lycopene, lutein, beta-cryptoxanthin, zeaxanthin, and astaxanthin. The antioxidant actions of carotenoids are based on their singlet oxygen quenching properties and their ability to trap peroxyl radicals [46, 47]. Flavonoids (i.e., quercetin) and phenolic acids (i.e., resveratrol) are polyphenolic substances that have chemical structures supporting the scavenging of free radicals and the chelation of redox-active metals [48].

## 3. Antioxidant Therapy

Antioxidant therapy has been defined as any treatment that prevents or decreases the adverse effects of oxidants. One of the strategies for pharmacological modification of oxidant-mediated tissue injury focuses on increasing the antioxidant capacity of cells or preventing the generation of ROS [12, 13]. The effectiveness of exogenous antioxidants to protect tissues from oxidant stress *in vivo* depends on the antioxidant used, its physicochemical and biopharmaceutical properties, its availability at the site of action, and, the nature of the oxidant stress [12, 13]. 

Only a few available drugs have been documented to possess antioxidant properties that might contribute to their efficacy. For example, N-acetylcysteine is used as a mucolytic agent and in the treatment of acetaminophen poisoning [86], deferoxamine is used as a chelating agent in iron overload [87], and probucol, calcium antagonists, *β*-adrenoreceptor antagonists, antiarrhythmic drugs, and statins are used in the treatment of cardiovascular diseases [88, 89]. Most of the studies examining the prophylactic or therapeutic effects of antioxidants against several diseases and disorders have resulted from epidemiological studies in ethnic groups who have different lifestyles and have been exposed to different environmental factors [13]. For example, the use of red wine in the diet of French people is associated with lower incidences of cardiovascular disease [13]. Also, the high level of antioxidants in the Mediterranean diet has also been associated with lower incidences of cardiovascular disease and morbidity and mortality [13]. Other studies have focused on the use of antioxidants by patients as dietary supplements in the hope of maintaining health, preventing disease, or reducing the toxicity of chemotherapy and radiotherapy, but the results so far are not conclusive [12, 13, 90].

The benefit of dietary antioxidants has been explored in both *in vitro* and *in vivo* systems. Most antioxidants, when applied directly and at relatively high concentrations to cellular systems *in vitro*, are effective in conferring protection against oxidant insults. On the other hand, results from studies in animals and humans have revealed that several antioxidants provide only modest benefit and have yet to be rendered into reliable and safe antioxidant therapies. For example, exposure of animals to the antioxidant enzymes superoxide dismutase and catalase has been encountered with antigenicity and immunogenicity problems [91, 92], and both of these enzymes are poorly absorbed from and rapidly degraded in the gastrointestinal tract [13]. Other studies have shown that flavonoids possess potent antioxidant activity *in vitro*, but their antioxidant action *in vivo* has been challenged by studies examining the bioavailability of flavonoids, which indicate that they reach only very low concentrations in human plasma after the consumption of flavonoid-rich foods [93]. In addition, most flavonoids are extensively metabolized *in vivo*, which can affect their antioxidant capacity [93]. It is obvious that the failure of antioxidants to seriously modify the injurious actions of oxidants is primarily attributed to their physicochemical characteristics and/or pharmacokinetic properties without excluding other possibilities such as some antioxidants also have prooxidant activities, particularly in the presence of transitional metals [94–97]. Experimental evidence, therefore, supports the need for the development of formulations that would enhance the delivery and retention of antioxidants in the tissues. Strategies to improve the effectiveness of antioxidants are focused on chemical modifications of antioxidants (e.g., attachment of masking pegylated (PEG)-groups), coupling of antioxidants to affinity carriers (e.g., antibodies against cellular adhesion molecules) and drug-delivery systems such as micelles and liposomes [4, 13–15, 17]. In this paper, we will address the role of liposomes as a delivery system for antioxidants. 

## 4. Liposomes

**Figure 2:**
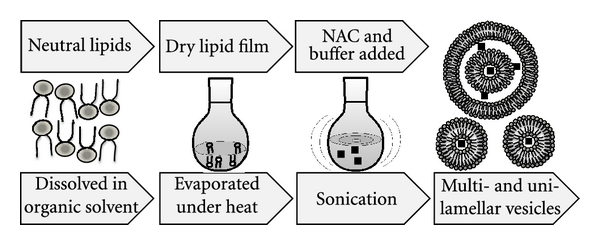
Preparation of liposomal-N-acetylcysteine (L-NAC). L-NAC is prepared from a mixture of DPPC and NAC in a 1 : 1 molar ratio by a dehydration-rehydration method. The lipids are dissolved in chloroform in a round-bottomed flask and dried at 45°C with a rotary evaporator. The lipid film is dried with nitrogen to eliminate traces of chloroform and hydrated with a solution of NAC and subsequently sonicated. Sonication is a simple method for reducing the size of liposomes. Upon rehydration, free NAC is separated by high-speed centrifugation (24400 g at 4°C, for 30 min), a step performed twice. At the end of this procedure, the liposomal vesicle size is usually below 200 nm mean diameter with an encapsulation efficiency of 35% NAC.

Liposomes are nanosized artificial vesicles of spherical shape that can be produced from natural or synthetic phospholipids. The polar head groups are located at the surface of the membranes, in contact with the medium, whereas the fatty acid chains form the hydrophobic core of the membranes, shielded from the water. Polar molecules can be encapsulated in the aqueous core, while hydrophobic molecules dissolve in the bilayers of liposomes [19]. Lipid film hydration is the simplest method for the encapsulation of water-soluble drugs; a lipid film is hydrated with an aqueous solution containing the drug (Figure 2). Entrapment efficiency may vary depending on: (i) the compound itself (charge, size, solubility, etc.), (ii) the lipid variations (a single type or a mixture of lipids, composition, charge, etc.), and (iii) the preparation methods. The manipulation and design of liposomes endowed with the ability for targeting specific cell sites (or alternately, the temporary avoidance of these sites), results in long circulation, increased biodistribution, and favourable pharmacodynamics [98–100]. Currently, a number of liposome formulations are in clinical use to combat cancer and infectious diseases, while others await clinical trial outcomes. For example, while DaunoXome, Doxil, and Ambisome are currently clinically approved, CPX-1 and LE-SN38 are examples of liposomal-based drugs that encapsulate a topoisomerase I inhibitor and are currently in Phase-II clinical trials for the treatment of colon or colorectal cancer [100]. Some of the setbacks and disadvantages of liposomes include time-consuming preparation techniques, low entrapment volumes, and toxicity concerns due to the presence of residual toxic organic compounds during preparation [98].

The lipids used for the preparation of liposomes are predominantly phospholipids or surfactants which form bilayers similar to those found in biological membranes. The surfactants dimyristoyl-phosphatidyl-glycerol (DMPG), dimyristoyl-phosphatidylcholine (DMPC), dipalmitoyl-phosphatidylcholine (DPPC), and desaturated-phosphatidylcholine (DSPC) are naturally occurring but can be produced synthetically as well. Extensive testing of these phospholipids has revealed them to be remarkably safe for pharmaceutical use. For example, phosphatidylcholine (i.e., DPPC) (a phospholipid that carries no net charges, is the major constituent of cell membranes, and it provides a structural framework for the membrane and maintains the permeability barrier) is well tolerated in animal and human studies. Inhaled nondrug containing liposomes (15 and 150 mg of lipid DPPC/mL) for 1 hour on pulmonary function and on oximetry in healthy nonsmoking volunteers showed that liposome inhalation is well tolerated, and no oxygen desaturation, decrements in pulmonary function, or side effects were noted [101]. Other investigators have utilized fluorescein-labelled liposomes to examine the clearance of aerosolized liposomes from the lungs of human volunteers and reported no untoward side effects [102]. Intravenous administration of DPPC (5 to 50 mg of lipid) did not induce immediate or delayed toxicity in mice and did not produce any changes in body weight and weight of major organs 2 weeks after administration. It has been reported that the toxicity of intravenously administered liposomes composed of DPPC is so low that accurate assessment of an LD50 value is difficult and has been estimated to be of the order of 10 g/kg in mice [103]. However, it is important to note that addition of other constituents to liposomes in order to alter stability or kinetics can result in an increase in toxic potential, particularly on parenteral administration of liposomes [104]. 

A potential problem with conventional liposomes, particularly when delivered intravenously, is their rapid removal from circulation by cells of the reticuloendothelial system (RES) particularly in the liver and spleen [14, 105]. To circumvent the phagocytic cells of the immune system and hence enhance their half-life in the circulation, “stealth liposomes” have been designed [14, 105]. Stealth liposomes are created by coating the liposomes with a layer of polyethylene glycol-phosphatidylethanolamine (PEG liposomes). PEGylation is the process of covalent attachment of polyethylene glycol polymer to another molecule masking the agent from the host's immune and metabolic systems and creating a shield around the pegylated agent due to its large hydrodynamic volume, thus protecting it from renal clearance and consequently prolonging its circulatory time [14, 105, 106].

## 5. Liposomes as an Antioxidant Delivery System

Liposomes have been considered to be excellent models of cell membranes and have been used for the evaluation of the antioxidant properties of several lipophilic and hydrophilic antioxidants against oxidant insults. As a drug delivery system, liposomes have been used for the transport of water-soluble and lipid-soluble antioxidants as well as antioxidant enzymes to different organs and tissues for the treatment of oxidative stress-induced damage. Up-to-date, delivery of molecules with antioxidant properties to different organs and tissues include the lipophilic antioxidants *α*-tocopherol [107, 108] and CoQ10 [13, 109, 110], the hydrophilic antioxidants glutathione, N-acetylcysteine [64, 111, 112], and quercetin [13, 83, 113], and the antioxidant enzymes superoxide dismutase and catalase [4, 14] (Table 1). Liposomes can facilitate intracellular delivery of several therapeutic agents via fusion with the plasma membrane lipids, receptor-mediated endocytosis, and phagocytosis [114–116]. 

### 5.1. Liposomal Superoxide Dismutase (SOD) and/or Catalase (CAT)

The effectiveness of the antioxidant enzymes, SOD and CAT, in the treatment of oxidant-induced injuries is limited because of their unfavorable physicochemical properties [13, 91, 92, 117, 118]. SOD is unable to cross cell membranes due to its high molecular mass (which prevents intracellular transport) or its charge (which prevents its adherence to targets) and possess immunogenic properties [15, 16, 91]. To enhance their effectiveness, polyethylene glycol was attached to antioxidant enzymes (pegylated SOD and/or pegylated CAT), an approach shown to increase their in vivo half-lives, cellular uptake by endothelial cells, and effectiveness in preventing pulmonary oxygen toxicity in rats [119, 120]. However, when compared to liposomal entrapped SOD and CAT, PEG antioxidant enzymes are less protective against lung injury from continuous hyperoxia; endothelial cells treated with liposomal entrapped SOD and CAT increase the activity of these enzymes by 44-fold within 2 hours [119, 121]. Another strategy involves the conjugation of SOD and CAT with an antibody to platelet-endothelial cell adhesion molecule-1 (PECAM-1) which bind to endothelial cells and alleviate oxidative stress in cell culture models as well as a mouse model of vascular oxidative stress [122–124]. Such validated strategies may be used effectively to develop future liposomal therapeutics by conjugating antibodies and other molecules to liposomes in order to provide specific targeting to endothelial cells or any other cell type [125, 126].

The pharmacokinetic profile of the antioxidant enzymes also provides hindrance in their effective use in the treatment of oxidant injuries. Intravenously administered SOD shows a biological half-life of a few minutes, and enteral administration is ineffective due to biodegradation of the enzyme in the gastrointestinal system [15, 16, 118]. On the other hand, systemic administration of liposomally encapsulated superoxide dismutase and catalase prolongs their circulating half-life and enhances their protection in animal models of oxidant-induced organ injuries [49, 57, 58]. For example, the short half-lives of native SOD and catalase in circulating blood and their inability to cross the blood-brain barrier limit their therapeutic usefulness in treating ischemic brain injury. Intravenous administration of Cu, ZnSOD liposomes facilitates the delivery of SOD into the brain, not only in the infarct but also in the noninfarcted subcortical area, resulting in significantly elevated levels of SOD activity and protection against cerebral ischemia/reperfusion injury [53–55]. Also, the addition of polyethylene glycol to the surface of the liposomes gives the liposomes a hydrophilic “sterically stabilized” surface, a property that contributes to a lower affinity of macrophages of the mononuclear phagocyte system (MPS) for the circulating liposome particles and consequently to an even extended prolonged blood circulation (more than 5-fold prolongation of liposome circulation time in blood) [127]. Systemic administration of PEG-liposomes for targeting SOD to arthritic sites with that of non-PEG-liposomes containing stearylamine in rats with adjuvant arthritis demonstrate that PEG-liposomes are superior with respect to circulation time and extent of localization at arthritic sites [50]. 

Intratracheally administered surfactant liposomes, encapsulating CuZn-superoxide dismutase and catalase, increases the alveolar type II cell antioxidant activity and protects cells against oxidant stress in the lungs of adult, premature or newborn animals [59–61, 65]) or against bleomycin-induced lung injury [62, 63]. The coinstillation with 2-chloroethyl ethyl sulfide (CEES) of liposomes containing pegylated- (PEG-) catalase (CAT), PEG-superoxide dismutase (SOD), or the combination, greatly attenuated the development of lung injury [64]. Results from a limited number of clinical studies suggest that liposomal superoxide dismutase might protect against radiation-induced fibrosis [51, 52]. The biological half-life of recombinant human Cu/Zn SOD (rhSOD) within the systemic circulation by liposomal encapsulation and aerosolization into the lungs of pigs leads to long-term and uniform uptake into systemic circulation without acute deleterious effects on respiratory tract suggesting that aerosolization of liposomal rhSOD could be a feasible strategy for administration of radical scavenging enzymes for treatment of systemic diseases [128]. 

The local application of liposome-encapsulated SOD has been shown to confer beneficial effects as well. Subgingival application of liposome-encapsulated SOD with scaling and root planning suppressed periodontal inflammation on experimentally induced periodontitis in beagle dogs, a treatment effect attributed to the slow washing of the formulation from the periodontal pocket due to the good bioadhesive properties of the vehicle [56]. Topical application of cationic liposome-encapsulated SOD to rat jejunum prior to induction of oxidative injury *in situ* was found to significantly enhance the antioxidant effect of SOD against the induced oxidative damage in the jejunal mucosa, compared with their free forms; this effect was caused by the increased mucosal adhesion of the liposomes which potentially provide protection for the SOD against proteolysis and premature scavenging by mucin components [129]. In another study, topical application of liposomal-encapsulated SOD reduced postburn wound size and edema formation for the reason that liposomes enhanced the antioxidant effect of SOD against the neutrophil-mediated oxidant injury by promoting the controlled sustained release of SOD at the site of injury [130].

### 5.2. Liposomal Glutathione

Glutathione in its reduced form (GSH) is the most powerful intracellular antioxidant. Decreases in circulating and intracellular concentrations of GSH levels can result in a lowered cellular redox potential influencing the translocation of the transcription factor NF kB which regulates the synthesis of cytokines and adhesion molecules [131]. One possibility to protect cells from damage caused by reactive oxygen species is to restore the intracellular glutathione levels. Cellular GSH concentration can be influenced by exogenous administration of GSH (as intravenous infusion or as aerosol) or glutathione esters [131]. The systemic bioavailability of orally administered GSH and glutathione monoethyl ester (GSHE) in the rat is low without affecting the circulating concentrations of GSH and cysteine, while intravenous administration of GSH or its ester results in rapid elimination albeit slower for the GSHE [132, 133]. Dietary glutathione is not possible to increase circulating glutathione to a clinically beneficial extent by the oral administration of a single dose of glutathione (3 grams) because of hydrolysis of glutathione by intestinal and hepatic gamma-glutamyltransferase [134]. However, consumption of liposomal glutathione (50 mg/kg/day) by atherosclerotic apolipoprotein E-deficient (E^0^) mice (which develop atherosclerotic lesions with many features common with human lesions) for 2 months significantly reduced serum and macrophage oxidative stress, macrophage cholesterol mass, and as a result of these effects, significantly attenuated atherosclerosis development when compared to mice that consumed similar concentration of control liposomes [66]. In another study, glutathione injected intravenously 2 hours before acetaminophen administration as a liposomal formulation was more effective than soluble GSH in protecting against drug-induced liver necrosis lipid peroxidation, and hepatic glutathione depletion [67]. 

In animal studies where GSH is administered intratracheally, only 1%-2% of the dose administered remained in the lung 24 hours after treatment, while liposome encapsulation improved the pulmonary retention of GSH, with 18% and 10% of the dose administered remaining in the lung 24 h and 48 h after treatment [135, 136]. Accordingly, Suntres and Shek [71] showed that intratracheal instillation of liposome-entrapped GSH yielded a better protection than free GSH against paraquat-induced lung injury. The improved protection conferred by the liposomal GSH formulation was attributed to the extended retention of liposomes in the lung, thus allowing a slow release of its GSH content [71]. In another study, Smith et al. [136], showed that intratracheal instillation of liposomal GSH was better than free GSH in protecting against hyperoxia-induced lung injury [136]. Intratracheal liposomal glutathione instillation in ventilated preterm infants raised the pulmonary glutathione and significantly reduced the levels of lipid peroxidation products [68]. 

It has been shown that liposomal GSH can be utilized for repletion and maintenance of intracellular GSH in neuronal cells and that liposomal GSH can provide significant protection to neurons in a model system relevant to Parkinson's disease [69]. Neurons, like most other cells, do not possess transport mechanisms for GSH and elevation of extracellular GSH may pose potential toxicity problems that increase neuronal vulnerability during ischemia, while the encapsulation of GSH into lipid vesicles may avoid the potential toxicity to neurons associated with extracellular GSH elevation and may facilitate drug delivery to cells as has been shown for other liposomal preparations [137, 138]. The concentration needed for half maximal repletion in mixed mesencephalic cultures containing approximately 70% neurons and 30% glia was 100-fold less when GSH was encapsulated into liposomal vesicles (4.75 *μ*M for liposomal GSH versus 533 *μ*M for nonliposomal fully reduced GSH) [69].

## 6. Liposomal N-Acetylcysteine

N-acetylcysteine (NAC), a thiol-containing compound, when administered in its conventional form did not protect against the prolonged shock-induced acute lung injury but when administered as a liposomal formulation directly to the lungs of animals protected against the lung injury [72]. The protective effect conferred by the liposomal NAC occurred at low NAC doses [72], since liposomes are known to prolong the retention of the antioxidant in the lungs. L-NAC was also shown to have a prophylactic effect against both LPS-induced lung injuries [112] and LPS- or acetaminophen-induced hepatotoxicity in animals [73, 111]. N-Acetylcysteine is a drug used in the clinic for the treatment of acetaminophen-induced hepatotoxicity and as a mucolytic agent. It possesses free radical-scavenging properties [4, 139] which are attributed to the nucleophilicity and redox interactions of its thiol group [139, 140]. Additionally, NAC is a source of cysteine, often the limiting precursor of *de novo* GSH synthesis [139, 141, 142]. Furthermore, NAC has been shown to influence redox-sensitive cell-signaling and transcription pathways, such as NF-*κ*B (which regulates pro-inflammatory genes), and the p38, ERK1/2, SAPK/JNK, c-Jun, and c-Fos pathways, among others, in a wide variety of different systems [139, 143]. N-Acetylcysteine has been shown to promote cell growth and survival by activating the MAPK pathway in response to ROS-induced injuries (which normally lead to growth arrest and apoptosis) and is able to limit inflammatory processes, such as the release of pro-inflammatory cytokines [144]. 

The use of L-NAC was also investigated in half sulfur mustard-induced acute lung injury in rats [64, 70] and guinea pigs [108]. McClintock et al. [64] and Hoesel et al. [70] found that the effects of airway instillation of 2-chloroethyl ethyl sulfide (CEES), which has been shown to induce acute lung injury (assessed by the leakage of plasma albumin into the lung) in rats, were attenuated with immediate and 1-hour-delayed instillation of liposomes containing reducing agents (i.e., NAC, GSH, or resveratrol) or bifunctional liposomes (containing NAC and GSH). Additionally, Hoesel et al. [70] found that airway instillation of L-NAC was protective when administered 4 hours following CEES application in rats, as well as 3 weeks after CEES with bifunctional *α*-tocopherol and NAC liposomes, though not with L-NAC alone after 3 weeks. Also, the intratracheal administration of liposomal antioxidant formulation containing alpha/gamma-tocopherol alone or with NAC immediately after instillation of CEES attenuated the short-term as well as long-term (fibrotic) effects of CEES-induced lung injury [64]. 

Irrespective of the route of administration, liposomal NAC was far superior to the conventional NAC. It has been shown that intravenous administration of NAC alone at a dose of 25 mg/kg was not effective in conferring any significant protection against LPS-induced hepatotoxicity while an equivalent dose of NAC delivered as a liposomal formulation conferred protection. This is not surprising, because most studies have shown that intravenous administration of conventional NAC preparations are demonstrated to be effective against animal models of sepsis or endotoxemia at doses greater than 50 mg/kg, iv [145–148]. Results from pharmacokinetic studies have shown that following intravenous administration, NAC undergoes rapid and extensive metabolism in the liver resulting in bioavailability of about 10% [149, 150]. This finding further supports the role of liposomes in their use as a drug delivery system because of their capability of delivering hepatoprotective agents to the liver, an ideal approach to increase local concentration of the agent, to reduce adverse effects, and to achieve maximal therapeutic efficiency. In a separate study examining the safety and pharmacokinetics of L-NAC in control rats, it has been demonstrated that the half-life of intravenously administered L-NAC is significantly increased by 4-fold from 6 min to 30 min (unpublished observation). Similarly, results presented by other investigators have shown that intravenous administration of drugs as liposomal formulations prolong their circulation time in blood and increase their distribution to major organs, including the lung [4, 13]. 

### 6.1. Liposomal Tocopherols

*α*-Tocopherol, the major component of Vitamin E, is a lipid-soluble hydrocarbon compound that partitions into lipid storage organelles and cell membranes. It is an efficient scavenger of lipid peroxyl radicals and, hence, it is able to break peroxyl chain propagation reactions in cellular membranes preventing lipid peroxidation [151, 152]. In studies with model membrane systems, *α*-tocopherol intercalates into phospholipid bilayers with the long axis of the molecule oriented parallel to the lipid hydrocarbon chains, and it is able to rotate about its long axis and diffuse laterally within fluid lipid bilayers [152, 153]. Because of its membrane stabilizing effect, *α*-tocopherol has been used in the preparation of liposomes for the delivery of several drugs [84, 154]. The maximum amount of *α*-tocopherol that can be contained in egg phosphatidylcholine or phosphatidylcholine liposomes is approximately 33% *α*-tocopherol. *α*-Tocopherol alters the membrane characteristics of liposomes by making them more stable and less permeable to aqueous solutes and highly resistant to protein-induced disruption [155]. The suppression of protein-induced disruption is more pronounced with *α*-tocopherol than with cholesterol (used in liposomal preparation to increase the physical stability of liposomes particularly in the presence of biological fluids such as plasma), even at lower molar ratios. Also, *α*-tocopherol in liposomes can undergo spontaneous intermembrane transfer to an acceptor membrane without the fusion of the with *α*-tocopherol liposomes [156]. Thus, liposomes containing *α*-tocopherol (15 to 30 mol%) may be useful for delivering physiological quantities of this vitamin component or other drugs to cells in culture or to tissues *in vivo*. 

Many studies have clearly demonstrated the important role of *α*-tocopherol in modulating oxidant-induced cellular injury, permitting cells with high levels of the antioxidant to become more resistant to oxidative insults. It has been shown that the administration of vitamin E, prior to an oxidative challenge, reduces the level of lipid peroxidation in several tissues and improves survival [152, 157–159]. However, in contrast to our studies [4, 74–78, 160], where the intratracheal instillation of *α*-tocopherol liposomes conferred a significant protective effect against the acute lung injuries and inflammation induced by several chemicals including paraquat, bleomycin, phorbol myristate acetate, and lipopolysaccharide (LPS), the oral or parenteral administration of conventional *α*-tocopherol to animals offered limited or no protection against paraquat or lipopolysaccharide-induced lung damage [161–165]. In our studies, the intratracheal instillation of *α*-tocopherol liposomes achieved a substantially higher antioxidant level in the lung, approximately 1 mg/g lung weight [166], while the amount of antioxidant recovered from the lungs of animals after oral or parenteral administration of vitamin E was most likely less than 40 *μ*g/g lung tissue [167, 168]. Vitamin E, used as oral supplements, is often in the form of tocopheryl esters which are highly stable to oxidation but are absorbed only after they have been unesterified by the intestinal esterases. The active form of *α*-tocopherol is highly viscous oil, practically insoluble in water and readily oxidized by atmospheric oxygen. In our laboratory, attempts were made to deliver *α*-tocopherol to the lungs of animals by other means, but results from these studies showed that organic solvents, such as ethanol and dimethylsulphoxide, used to solubilize the viscous free *α*-tocopherol, were toxic to the lung, and emulsifying agents and detergents, such as polyethylene glycol and Tween 80, caused respiratory failure, possibly by disrupting surface tension of the lung [166]. An intravenous form of vitamin E, E-ferol, that contained dl-(*α*)-tocopherol and polysorbate 80 was first clinically used, albeit without detailed safety tests, in the early 1980s in low-birth-weight and premature infants for the treatment of the retrolental fibroplasias and resulted in severe adverse effects, including hepatomegaly and renal failure most likely due to the excipient polysorbate 80 [169].

The effectiveness of *α*-tocopherol-loaded liposomes has been examined in tissue injuries other than the lung as well. Sinha et al. 2001 [80] showed that *α*-tocopherol-loaded liposomes were protective against an experimental in vivo rat model of global cerebral ischemia controlling more effectively the conjugated diene increment in the brain of rats after ischemia and reperfusion of rats [80]. Liposomal *α*-tocopherol formulations have been proven to be effective in tissue injuries induced by alkylating agents such as melphalan, 2-chloroethyl ethyl sulfide (CEES), [79, 108].

## 7. Other Liposomal Antioxidants

### 7.1. Liposomal Coenzyme Q

Coenzyme Q (CoQ) is a naturally occurring compound also known as ubiquinone. It belongs to a homologous series of compounds that share a common benzoquinone ring structure but differ in the length of the isoprenoid side chain; in humans and a few other mammalian species, the side chain is comprised of 10 isoprene units, hence it is called coenzyme Q10 (CoQ10). CoQ10 has a fundamental role in cellular bioenergetics as a cofactor in the mitochondrial electron transport chain (respiratory chain) and is, therefore, essential for the production of ATP. CoQ10 in its reduced form as the hydroquinone (called ubiquinol) is a potent lipophilic antioxidant and is capable of recycling and regenerating other antioxidants such as tocopherol and ascorbate [170, 171]. Data from several laboratories have demonstrated that CoQ10 pretreatment protects the myocardium from ischemia-reperfusion injury via both antioxidant and bioenergetic pathways [110]. However, the high molecular weight and lipophilicity of CoQ10 makes it poorly water soluble and consequently leads to low systemic availability [18]. Following oral administration of CoQ10 either as a powder or as an oil suspension elicit practically no response in human subjects and it takes days to increase the CoQ10 blood and possibly other tissue [109, 170]. The intravenous administration of CoQ10 as a liposomal formulation raised the serum and myocardial levels of CoQ10 and significantly improved the function and efficiency of myocardial tissue and reduced oxidant injury observed following ischemia-reperfusion [109]. Several advancements have been made to enhance the bioavailability of CoQ10 using various approaches like size reduction, solubility enhancement (by solid dispersion, prodrug, complexation, and ionization) and use of novel drug carriers such as liposomes, microspheres, nanoparticles, nanoemulsions, and self-emulsifying system (see review by Beg et al. 2010 [18]). 

Liposomal CoQ10 is a promising candidate for the topical application of CoQ10. CoQ10 encapsulated in N-trimethyl chitosan- (TMC-) coated liposomes is being examined as potential ophthalmic drug delivery system. TMC exhibits an excellent absorption-enhancing effect by opening the tight junctions between adjacent cells of epithelial cell monolayers [172] while Q10 ameliorates the oxidative stress in the lenses, the most common damaging factor for the development of cataract [173]. Also, cosmetically applied CoQ10 has the ability to reduce photoaging, with a corresponding decrease in wrinkle depth [84], but its bioavailability in skin is poor. A liposomal formulation composed of soybean phosphatidylcholine (SPC) and alpha-tocopherol (Vit E) used to encapsulate CoQ10 for topical application significantly enhanced its accumulation (at least twofold) in rat skin, compared with an unencapsulated suspension. Prolonging the treatment time and increasing the content of CoQ10 in the formulation both raised the amount of CoQ10 in rat skin [84].

### 7.2. Liposomal Curcumin

Curcumin, a hydrophobic polyphenolic compound derived from dietary spice turmeric, possesses diverse pharmacologic effects including anti-inflammatory, antioxidant, antiproliferative, and antiangiogenic activities. Curcumin has been used extensively in traditional medicine since ancient times as a household remedy against various diseases, including hepatic disorders, cough, sinusitis, rheumatism, and biliary disorders. In the past few decades, a number of studies have been carried out to confirm curcumin's potential role in treating inflammatory disorders, cardiovascular disease, cancer, AIDS, and neurological disorders [174–176]. The main drawback associated with the therapeutic potential of curcumin is its poor aqueous solubility and stability in gastrointestinal fluids, which leads to poor bioavailability [175, 176]. Several novel drug-delivery approaches, including microemulsions, nanoemulsions, liposomes, solid lipid nanoparticles, microspheres, solid dispersion, polymeric nanoparticles, and self-microemulsifying drug-delivery systems have been used to enhance the bioavailability and tissue-targeting ability of curcumin *in vitro* and *in vivo* [175–177]. Oral administration of liposomal curcumin increased its bioavailability and antioxidant activity in plasma [85]. A recent study examining the antitumor activity of liposomal curcumin against human pancreatic carcinoma cells demonstrated that liposomal curcumin inhibits pancreatic carcinoma growth and, in addition, exhibits antiangiogenic effects [178].

### 7.3. Liposomal Resveratrol

Resveratrol, the main active polyphenol in red wine, has been implicated as a possible contributor to the cardiovascular protection conferred by red wine consumption, since this compound can be found in grapes among other fruits [179, 180]. The cardiovascular effect of resveratrol can, in turn, be related with its proved capacity to act as a modulator on the metabolism of lipoproteins inhibiting the oxidation of low-density lipoproteins (LDLs), and inhibiting either platelet aggregation or proatherogenic eicosanoids production by human platelets and neutrophils. These potential therapeutic and prophylactic applications are, however, restricted by the low bioavailability caused by its physical properties [179, 180]. Resveratrol has low water solubility and stability as well as its is rapidly and extensively metabolized making its clinical use challenging [181]. To our knowledge, there have been only a few studies comparing the antioxidant properties of liposomal resveratrol with that of free resveratrol and reported that the liposomal formulation is more effective in ameliorating the oxidant-induced cellular injury induced from UV-B irradiation on human-derived renal epithelial cells [182].

### 7.4. Liposomal Quercetin

Quercetin is a polyphenolic flavonoid, commonly found in apples, onions, berries, and red wine, with strong antioxidant and antiinflammatory properties. Its use is limited by its low water solubility and high rate of metabolism [183]. Pretreatment of animals with the flavonoid quercetin was ineffective in protecting against carbon tetrachloride-induced hepatotoxicity. Carbon tetrachloride is known to mediate its liver toxicity via free-radical mechanisms including oxidative stress. Pretreatment of animals with mannosylated liposomal quercetin significantly lowered the hepatotoxic effect of carbon tetrachloride [83]. In another study, it was demonstrated that quercetin-filled liposomes improved the protective effects of the antioxidant against peroxynitrite-induced myocardial injury in isolated cardiac tissues and anesthetized animals [82]. Pegylated liposomal quercetin significantly improved the solubility and bioavailability of quercetin and enhanced its antitumour activity in immunocompetent C57BL/6N mice bearing LL/2 Lewis lung cancer and in BALB/c mice bearing CT26 colon adenocarcinoma and H22 hepatoma [113].

### 7.5. Liposomal Astaxanthin

Astaxanthin, is a red-orange lipid-soluble carotenoid pigment with powerful antioxidant and antinflammatory properties. As measured by a fluorometric assay based on visible-absorbing fluorescent probes that belong to the BODIPY class of dyes (BODIPY 581/591 C_11_ and BODIPY 665/676) to measure antioxidant activities of antioxidants in a lipid environment, astaxanthin possess an antioxidative capability 10-times stronger than zeaxanthin, lutein, canthaxanthin, and *β*-carotene [184]. However, its use is often limited due to its poor water solubility and extremely low bioavailability when administered orally. The transport and protective effects of astaxanthin in Hep3B and HepG2 cell lines challenged with gamma radiation was far superior when the antioxidant was delivered with egg-yolk phosphatidylcholine liposomes [185]. Incorporation of astaxanthin into phosphatidylcholine multilamellar liposomes strongly reduced lipid damage when the lipoperoxidation promoters—H_2_O_2_, *tert*-butyl hydroperoxide (*t*-ButOOH) or ascorbate—and Fe^2+^:EDTA were added simultaneously to the liposomes [186] but also liposomal encapsulation is also effective in slowing the oxidation of astaxanthin [185].

## 8. Liposomes Containing More than One Antioxidant

It has been shown that the administration of liposomes containing more than one antioxidant is more advantageous in ameliorating oxidant-induced tissue injuries [57, 71, 80]. The antioxidant effect of liposomal formulations containing the enzymes SOD and catalase is more effective than those containing the individual antioxidant enzyme [57, 58]. The therapeutic efficacy of an antioxidant liposome formulation, containing a lipophilic antioxidant, *α*-tocopherol, can be improved by encapsulating another antioxidant, such as GSH or L-ascorbic acid, in the same liposome preparation. These liposomes, containing both *α*-tocopherol and GSH were shown to be more effective in protecting against oxidant-induced lung injuries and lipid peroxidation than those containing either *α*-tocopherol or GSH alone presumably by *α*-tocopherol scavenging free radicals and stabilizing biological membranes, while GSH, in addition to its ability to act as a free radical scavenger, can also regenerate *α*-tocopherol from its oxidized form [71]. Similarly, liposomes containing both *α*-tocopherol and L-ascorbic acid were more effective in preventing the generation of conjugated diene in cerebral tissues by the induction of global cerebral ischemia and reperfusion than the those containing L-ascorbic acid through a synergistic action [80]. In a more recent study, it was shown that liposomes containing *α*-tocopherol, *γ*-tocopherol, *δ*-tocopherol, and NAC were more effective than liposomes containing only NAC or GSH in blocking the CEES-induced inflammatory response and lung injuries [81, 108]. Liposomal delivery of curcumin and resveratrol reduced cancer in prostate-specific PTEN knockout mice and this treatment effect was far superior to that seen following administration of each agent alone either in their free or liposomal form [187].

## 9. Concluding Remarks

Antioxidant liposomes hold great promise in the treatment of many diseases in which oxidative stress plays a significant role. Antioxidant enzymes and many of the other antioxidants do not easily penetrate the plasma membrane of cells and some have poor stability and short half-life in plasma when administered through conventional delivery modes. Liposomes are highly efficient in terms of facilitating antioxidant delivery and achieving prophylactic and therapeutic efficacies against oxidative stress-induced damage. Liposomes can be prepared from natural phospholipids that are biocompatible, biodegradable, and nonimmunogenic and practically do not cause toxic effects or antigenic reactions. Although successful clinical applications in the field of drug delivery and treatment of cancer and systemic or local fungal infections have demonstrated the potential of the technology, clinical applications for the utilization of liposomal antioxidant formulations have been limited. Controversy on the efficacy and safety of antioxidants still exists due to lack of well-controlled clinical trials. There is a need for large multicentre prospective randomized control trials to assess the effects of different types and doses of antioxidant in selected groups of patients in various chronic and acute oxidative stress-related diseases. Perhaps, it is time to vigorously seek alternative approaches and not simply continue to study the use of antioxidants by patients as dietary supplements in the hope of maintaining health and preventing disease. The ability for precise adjustments of liposome parameters such as size, charge, lipid composition, and the conjugation of ligands coupled with the more efficient and safer loading techniques offers the flexibility to make liposomes an effective platform for the delivery of antioxidants.

## Figures and Tables

**Table 1:** Antioxidants delivered as liposomal formulations in animal models of oxidative stress.

Antioxidant	Route of administration	Experimental model	Target organ	Reference(s)
	iv	Acetaminophen poisoning (rat)	Liver	[49]
	iv (PEG-Liposome)	Rheumatoid arthritis (rat)	Paws	[50]
SOD	im	Radiation-induced injury (human)	Skin and underlying tissues	[51, 52]
	iv	Cold-induced brain injury (rat)	Brain	[53]
	iv	Cerebral ischemia-carotid artery occlusion	Brain	[54, 55]
	subgingivally	Peridontitis (dogs)	Gingiva	[56]

	iv	Hyperoxia	Lung	[57, 58]
	it	Oxidative stress-induced injury (Xanthine/xanthine oxidase ) (rabbit)	Lung	[59]
SOD and/or CAT	it	Hyperoxia (rat)	Lung	[60]
	it	Hyperoxia (premature rabbit)	Lung	[61]
	it	Bleomycin-induced injury (rat)	Lung	[62, 63]
	It (PEG-Liposome)	2-chloroethyl ethyl sulfide (CEES) (rat)	Lung	[64]

CAT	it	Hyperoxia (rat)	Lung	[65]

	oral	Atherosclerosis (mice)	Aortic arch	[66]
	iv	Paracetamol poisoning (mice)	Liver	[67]
GSH	it	Human-premature infants	Lung	[68]
		Parkinson's disease	*In vitro* neuronal/glial cell cultures	[69]
	it	2-chloroethyl ethyl sulfide (CEES) (rat)	Lung	[64, 70]

GSH/*α*-tocopherol	it	Paraquat poisoning	Lung	[71]

	it	Shock (rat)	Lung	[72]
NAC	iv	Acetaminophen poisoning (mice)	Liver	[73]
	it	2-chloroethyl ethyl sulfide (CEES) (rats)	Lung	[70]

	iv	LPS-induced injury (rat)	Lung	[74]
	iv	LPS-induced injury(rat)	Liver	[75]
*α*-tocopherol	it	Bleomycin (rat)	Lung	[76]
	it	Paraquat poisoning	Lung	[77]
	it	Phorbol-myristate acetate (rat)	Lung	[78]
	ip	Melphalan toxicity (mice)	Lung	[79]

*α*-tocopherol/L-ascorbic acid	iv	Partial cerebral ischemia (rat)	Brain	[80]

*α*,*γ*,*δ*-tocopherols/NAC	it	2-chloroethyl ethyl sulfide (CEES) (guinea pigs)	Lung	[81]

Quercetin	intraventricular	Myocardial injury (rats) peroxynitrite-induced myocardial injury in isolated hearts and animals	Heart	[82]
	iv	Arsenite poisoning (rats)	Liver	[83]

CoQ10	topical	Photoaging	Skin	[84]

Curcumin	oral	Bioavailability study	Plasma	[85]

Iv: intravenous, It: intratracheal, Ip: intraperitoneal, Im: intramuscular, and PEG: polyethylene glycol.
